# Smart City Scenario Editor for General What-If Analysis

**DOI:** 10.3390/s24072225

**Published:** 2024-03-30

**Authors:** Lorenzo Adreani, Pierfrancesco Bellini, Stefano Bilotta, Daniele Bologna, Enrico Collini, Marco Fanfani, Paolo Nesi

**Affiliations:** DISIT Lab, Department of Information Engineering, University of Florence, 50139 Florence, Italy

**Keywords:** smart city, dashboard, what-if analysis, scenario editor

## Abstract

Due to increasing urbanization, nowadays, cities are facing challenges spanning multiple domains such as mobility, energy, environment, etc. For example, to reduce traffic congestion, energy consumption, and excessive pollution, big data gathered from legacy systems (e.g., sensors not conformant with modern standards), geographic information systems, gateways of public administrations, and Internet of Things technologies can be exploited to provide insights to assess the current status of a city. Moreover, the possibility to perform what-if analyses is fundamental to analyzing the impact of possible changes in the urban environment. The few available solutions for scenario definitions and analyses are limited to addressing a single domain and providing proprietary formats and tools, with scarce flexibility. Therefore, in this paper, we present a novel scenario model and editor integrated into the open-source Snap4City.org platform to enable several processing and what-if analyses in multiple domains. Different from state-of-the-art software, the proposed solution responds to a series of identified requirements, implements NGSIv2-compliant data models with formal descriptions of the urban context, and a scenario versioning method. Moreover, it allows us to carry out analyses on different domains, as shown with some examples. As a case study, a traffic congestion analysis is provided, confirming the validity and usefulness of the proposed solution. This work was developed in the context of CN MOST, the National Center on Sustainable Mobility in Italy, and for the Tourismo EC project.

## 1. Introduction

Thanks to the increasing deployment of Internet of Things (IoT) technologies and the availability of big data, the concept of a smart city is, nowadays, widely applied worldwide to address current and future challenges in the urban context. Indeed, due to increasing urbanization, city councils are called to plan and take actions in several domains like mobility [1,2], urban infrastructure [3], energy [4], security [5,6], environment [7], etc. For example, traffic congestion and related pollution emissions must be reduced, green and sustainable energy sources should be adopted, critical areas should be identified and monitored, and public spaces should be improved to be safer and more accessible. Smart city IoT platforms [8] with interactive dashboards and advanced urban digital twin interfaces [9,10] are fundamental tools for assessing the current and past states of cities since they can provide city operators and decision makers immediate access to relevant information. However, such technologies should be improved. Solutions for tactic and strategic planning with prediction and simulation capabilities have to be included to help decision makers in urban development. In particular, the introduction of what-if analysis solutions [11] is required to observe the impacts of possible changes in the current urban scenario and to offer decision makers effective decision support system (DSS) tools. Such solutions should provide a structured framework for data-driven decision-making processes. Leveraging advanced algorithms and real-time data integration, they allow the users to experiment and evaluate the impacts of changes in the urban environment. The first step is the introduction of variations into the representations of smart city entities (roads, buildings, services, green areas, etc.), usually modeled with ontologies or relational databases [12]. Therefore, a graphical interface for altering the current representation and formalizing a set of hypothetical scenarios, i.e., a scenario editor, is mandatory. The availability of such a tool would make it possible to study the effects of city policies with data-driven approaches in terms of Key Performance Indicators (KPIs) and metrics.

In the literature, it is possible to find solutions addressing the formalization of scenarios and corresponding editor tools mainly in the context of autonomous driving [13,14]. However, such solutions focus on system responses to specific conditions, e.g., complex maneuvers involving multiple vehicles. The formalization of the scenario is also useful in decomposing the problems for fog and edge computing [15,16]; for shaping the context for computations and actions [17]; for contextualizing and shaping the user behaviors [18], etc. To define a scenario, classic GIS (geographic information system) tools, e.g., QGIS (version 3.34.5) [19] and ArcGIS (version 10.8.2) [20], could be used. Such tools allow the definition of shapes over maps and IoT and points of interest (POIs) services, and other references can be manually loaded. However, GIS software (https://gisgeography.com/best-gis-software/) tools are typically very far from providing easy-to-use solutions for editing a road graph and the corresponding semantic information such as priorities, lanes, restrictions, velocities, etc. Thus, GIS tools are inadequate for producing scenarios to be exploited for simulation/computation since many other data have to be added manually. Even if standards for formalizing a scenario emerged—such as the ASAM OpenSCENARIO [21] used in conjunction with the ASAM OpenDRIVE [22] and OpenCRG [23] standards used to describe static and dynamic characteristics—more general tools and models that are able to cope with a wider concept of a city scenario seem to be less investigated. Some solutions with specific capabilities have been proposed as commercial or open-source software. For example, the OpenStreetMap (OSM) ecosystem provides the iD editor (version 2.28.0) [24], a web-based editor used to modify OSM map elements intuitively. However, the introduced changes are directly incorporated into the OSM database, limiting the possibility of producing multiple scenarios to be analyzed simultaneously. In using the OSM iD editor, the area of interest of a scenario needs to be extracted using complex SQL queries and cannot be formally defined or directly exploited for successive computations and/or simulations. More advanced models and tools have been proposed, such as by SUMO (version 1.19.0) [25], and by PTV with the Vissim (version 2024.00-05) [26] and Visum (version 2024.01-05) [27] simulators. SUMO is an open-source traffic simulator that includes the SUMO netedit tool as scenario editor [28]. SUMO netedit allows the addition, modification, and removal of roads and connections as well as the alteration of element attributes, such as the number of lanes, speed limits, etc. Different file formats are accepted as input and output, including the OpenDRIVE standard for simulators. PTV Vissim and Visum are two proprietary simulators, for micro and macro scales, respectively. Both solutions include a similar editor, where the user can define changes to the current scenario by altering the road network. However, both SUMO and PTV editors require on-premise installations, while a web-based interface would be preferable for easier access and wider distribution. Moreover, they present a limited flexibility since cannot automatically extract and integrate information coming from IoT sensors (e.g., time series), which are an important source of information typically available in smart city environments and can be used to extend the focus of the simulation from the traffic to other problems like pollution, waste management, index computation, etc.

In this paper, the development of a model for scenarios and the corresponding web-based open-source tool for scenario editing are presented. To this end, a collection of requirements (collected from the activities of the national centers of sustainable mobility in Italy, CN Most) have been identified and analyzed. From the scenario editor, the user can select an area of interest and modify the topology and the attributes of the road network. Then, different kinds of IoT devices, entities, and services can be recalled and selected to complete the formalization of the scenario for the analysis phase based on computation and machine learning or artificial intelligence (AI) solutions/simulation. The aim was the definition of a powerful scenario model and tool that can be used to characterize the context for a huge number of context-depended computational activities (in time and space) such as those for computing people flows, traffic flow reconstructions, heatmaps, origin–destination matrices, indicators, navigation, representation, etc. Information can be gathered from multiple big data spaces, knowledge bases/graphs, etc. The users are therefore able to produce hypothetical scenarios/contexts on which computations and simulations could be performed, as in a what-if analysis. The formalization of scenarios in a flexible manner enables the assessment of the impact of changes introduced in complex city contexts. Moreover, our modeled scenarios can change their status (e.g., proposed, approved, closed), evolve over time, and be shared among experts and decision makers. In the proposed scenario management, the model definition and structure are formalized as a smart data model compatible with the FIWARE NGSIv2 (Next Generation Service Interface, version 2) standard [29] for data exchanges in and indexing into a knowledge base for future retrieval through semantic queries.

The proposed solution improves on the state-of-the-art solutions by providing a more flexible tool able to cope with different domains (while available solutions are focused on transportation analysis). Moreover, it can automatically gather historical and real-time information without needing to import data manually and supports versioning and state evolution. This facilitates the production and usage of scenarios and enables collaborations among several operators/analysts. In addition, case studies are provided in which the scenario model and tool are used to define the context needed to compute the traffic flow reconstruction of a portion of the city, or heatmaps. Both actual condition analyses and what-if analyses with changed constraints are discussed. The proposed scenario model and editor were developed and integrated into the Snap4City platform (https://www.snap4city.org/ (accessed on 19 February 2024)). Snap4City is an open-source IoT platform able to manage multiple tenants and billions of data with a key focus on interoperability. An example of the integrated scenario editor is accessible as a dashboard [30]. The scenario editor was embedded as a novel module of the Dashboard Builder Multi Data Map widget [31] and can retrieve static (road graph, urban elements, sensor positions, etc.) and real-time (sensor readings, public transport time schedules, weather, temperature, etc.) data through specific APIs. 

In summary, the main contributions of this paper are as follows: (i) the definition of a series of requirements that a scenario editor must meet; (ii) the definition of the smart data model describing a scenario and a formal model to describe the road network; (iii) the development of an open-source web-based scenario editor, and its integration into the Snap4City platform; and (iv) case studies to show the scenario editor functionalities applied to the traffic flow reconstruction problem to validate the scenario model and tool.

This paper is organized as follows. In Section 2, the general architecture and data flow related to the scenario model and editor are presented. In Section 3, the requirements defined to guide the development of the scenario model and editor are provided, together with the formal definition of a scenario as a smart data model. In Section 4, the scenario editor is described, and the results of a usability test are reported. Section 5 presents case studies where the scenario model and editor are used to perform traffic flow reconstruction (TFR) and congestion analysis in a what-if context. Finally, in Section 6, conclusions are drawn. This research was performed in the context of the CN MOST, the National Center on Sustainable Mobility in Italy [32]; Snap4City is one of the reference platforms for the CN MOST.

## 2. Context Definition

**Figure 1:**
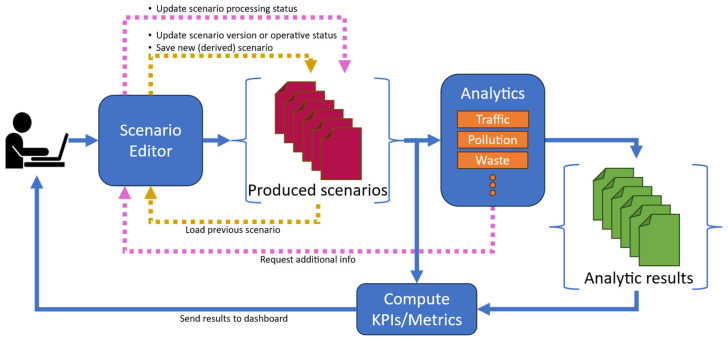
Block diagram describing the architecture of the scenario editor model, evolution, and exploitation.

Figure 1 depicts a conceptual block diagram describing the workflow for scenario production and evaluation, therefore defining the operative context for the scenario editor. As can be seen, the user interacts with the scenario editor interface by specifying the area of interest, date and time, metadata, loading and selecting real or simulated data (sensors, services), etc., as well as maybe altering the current scenario in all details, also in successive versions. All the data encompassed in the context of the scenario can be easily retrieved from Snap4City storage through visual or traditional queries on the Km4City knowledge base or any other storage. The produced scenario, with or without modifications with respect to the current condition, can be saved, exported/imported, and shared according to a smart data model (see Section 3.1). A scenario can be readily used as input to compute KPIs and metrics: for example, the computation of heatmaps based on sensor data in the scenario or the evaluation of the 15 min city index. Single or multiple scenarios can be used to contextualize (i.e., limiting in scope and parametrization) one or more data analytic processes to compute the TFR [33], heatmaps of pollutants or people flow, management of waste [34], etc. Analytic results may be passed to processes for KPI and metric computations to quantify the impact of the produced scenarios, and to compare the value of those KPIs/metrics in the current status and in the modified scenario according to the changes as in the what-if analysis. Finally, the results are obtained, and comparisons are shown to the user or saved for further analysis.

Note that the scenario editor can take as input a previously created scenario (see yellow arrows in Figure 1) that the user can further modify to obtain a novel version of the same scenario, create a new scenario derived from the old one, or change the operative status of the scenario, e.g., from *proposed* to *approved* or *rejected* statuses. This approach opens the path for collaborative works on what-if analysis, city development, and studies [35,36], and for the exploitation of generative AI. 

Some analytics may require additional information (pink arrows in Figure 1). In this case, the scenario is re-opened by the user to add/specify more information and changes, evolving the status of the scenario, e.g., from *init* status to *under analysis*. Using the scenario versioning and status, it is possible to save and track the history/evolution of a scenario and reduce the work of the users. To provide the reader an example, let us now suppose that due to scheduled street works, some roads should be closed to traffic; thus, a city mobility operator must find the best solution to avoid congestion, preserving viability. The mobility operator of the city has to assess the impact and find a solution, maybe among a possible set of options. They start by creating a first scenario, $S_{1}$, by closing some roads to traffic to study what would happen if those changes were performed. Then, $S_{1}$ is loaded into the editor, and the user adds further changes, such as inverting some road travel directions, creating a scenario $S_{2}$. The operator loads again $S_{1}$, and this time changes the number of lanes of some roads to create the scenario $S_{3}$.$\;S_{2},$ and $S_{3}$ are derived scenarios from $S_{1}$, and both have version set to *v*0 and operating status set to *proposed*. $S_{2}$ and $S_{3}$ are sent to the TFR analytic tool to assess which is the better solution to limit traffic congestion due to the closed roads. The city chief of the mobility operators decides that $S_{2}$ is not a valid solution: the operative status of $S_{2}$ passes to *rejected*. At the same time, the city council requires further changes to $S_{3}$ that the operator implements, updating the version of $S_{3}$ to *v*1. After a second round of KPI reconstruction and computation, $S_{3}$ is accepted, and its operative status moves to *approved*. Thus, the formal definition of a robust model for scenarios is the first step to create novel AI-based tools for the automatic generation of the best scenarios that can be verified and selected by the city mobility chief according to some KPIs.

## 3. Requirement Analysis and Scenario Data Model Definition 

In this section, the identified functional and non-functional requirements for the development of the scenario editor are presented and discussed. Functional requirements are reported in Table 1, with an ID, name, and brief description, while non-functional requirements are discussed in the following. 

As can be seen in Table 1, eleven main functional requirements have been defined. Since scenario editing is performed on geographical areas, a ground map and associated controls are required (R01). Requirements R02 to R04 are needed to define the specific context for the scenario. These include the drawing of the area of interest, the description of metadata, and the selection of the knowledge base or storage from where to fetch the data. Requirements R05 and R06 describe the main viewing and editing functionalities available to the user, from the selection of the data to consider to their manipulation to define alterations of the current scenario. Note that, according to R07, a scenario editor must allow users to load (and modify) heterogeneous data so as to be exploitable with multiple analytics and KPIs to perform different kinds of what-if analyses. Since the editor allows the operators to alter entities and roads as well as select different areas, a check of consistency (R08) should be carried out to avoid the creation of unrealistic scenarios: for example, unreachable road segments due to wrong travel direction assignments. Thus, it should be possible to create scenarios with different statuses and versions (R09): a processing status progression could be required by specific analytics, requiring user intervention at different steps of the process. On the other hand, the scenario’s operative status and version could be used to describe the evolution of the scenario, track the introduced changes, and maybe revert them. Therefore, the scenario editor should provide the possibility to create and save the scenario, to load a previously defined scenario (for example to define a new version or to advance the scenario status), and save it with a new name (R10). Finally, the scenario editor must produce a scenario conformant to a given model that should be sufficiently elastic to accommodate additional variables (R11).

Some non-functional requirements must also be satisfied. At first, the editor should be implemented as a web application to avoid the need for installing software and guarantee wide accessibility, coworking, and executing computing aspects on the cloud. A high performance level should be achieved in terms of fast response times to offer seamless interactivity, high reliability, and availability to avoid service interruption. Security and privacy aspects must also be considered: this requires the implementation of an adequate management of user profiles with different roles and with different organizations with which any user can be associated. Moreover, the possibilities of delegating or making data public, defining scenarios, or analyzing results are required to enable multiple users to work on the same problem collaboratively. For example, an operator could prepare different scenarios and results and then delegate/share them to the office manager/chief who makes the final decision.

To satisfy all the non-functional requirements and some of the functional ones (R01, R04, R05, R06, R07), the proposed scenario editor was integrated into the open-source smart city platform Snap4City. The platform includes functionalities for collecting/aggregating data from different sources through push and pull modalities using brokers, gateways, and services, and for indexing them in the Km4City knowledge base, as well as shadow storing the data in an OpenSearch cluster. In using specific APIs, data can be retrieved using spatial, temporal, and relational queries, therefore addressing R04, R05, and R06. Multiple analytical methods (R07) are available as well as solutions used to compute KPIs based on Node-RED flows according to national and international specifications like the SUMP [37], Italian PUMS [38], and European SUMI [39]. Moreover, Snap4City is GDPR [40]-compliant and successfully passed several penetration tests, uses multiple user roles, handles different organizations, and implements data and service ownership functionalities with the possibility to change ownership, delegate, or make the resources available for given organizations or to the public. The main installation uses up-to-date redundancy solutions to guarantee a high level of reliability. The proposed scenario editor was integrated as an extension of the multi-data map of the DashboardBuilder to create a map widget with navigation controls (R01) that can be included in any Snap4City dashboard and accessed with any web browser.

In Table 2, a comparison of state-of-the-art scenario editors is provided, checking their compliance with respect to the identified functional requirements reported in Table 1. As it can be seen from the summary table, GIS software [19,20] and the OSM iD editor [24] are the less compliant solutions. Even if they permit to load data and perform some manipulations on roads and entities, such solutions, for example, do not allow a clear definition of a scenario (R02, R03) and do not include analytics to perform analyses or simulations. The SUMO netedit tool [28] and the PTV products [26,27] are more advanced solutions. However, they also present some limitations; road graphs and entities can be imported, usually without ground maps (orthomap) (R01), and do not permit the definition of a specific area of interest, since it is implicitly defined by the user when importing the data (i.e., different data imports should be performed to work on different parts of a city). Moreover, they do not include the automatic retrieval of real-time data (R06) and are strictly focused on traffic analysis, limiting their applicability (R07). It should be noted that SUMO netedit executes a consistency check only during simulation and not when saving the scenario (R08), and scenario versioning must be manually performed by saving different files (R09). Contrarily, the proposed Snap4City scenario editor can satisfy all the requirements, offering an editor that can be exploited in different domains, with versioning support, etc. Moreover, concerning the non-functional requirements, except for the OSM editor, all the other state-of-the-art solutions require on-premise installations and do not offer security or privacy characteristics, such as a precise access control at the level of a single scenario.

### 3.1. Scenario Data Model

Here, a formal definition of the data model for a smart city scenario is provided, responding to requirement R11. Such a definition was developed to store the needed data and information according to the functional requirements presented in the previous section. A scenario is described as a context entity compliant with the FIWARE NGSIv2 specification [29], with a type *SmartCityScenario* and an ID defined as an URI, corresponding to an entity instance in the knowledge base. A scenario has the following attributes (with data types reported in brackets):

A1. *name* (string): the name of the scenario;
A2. *description* (string): a brief description of the scenario;
A3. *location* (string): the textual name of the geographic area considered;
A4. *startDatetime* (string): timestamp of the starting instant from which the scenario is valid, represented as string compliant with ISO 8601 [41];
A5. *endDatetimes* (string): timestamp of the last time instant for which the scenario is valid, represented as string compliant with ISO 8601 [41];
A6. *areaOfInterest* (geometry): a polygon describing the portion of the city over which the scenario is defined, represented in GeoJSON;
A7. *knowledgeBase* (string): the ID of the knowledge base used to fetch the data in the scenario, represented as a URI. It also identifies an organization or tenant in the multitenant Snap4City platform;
A8. *entities* (data structure): IoT devices or other urban entities (e.g., traffic sensors, semaphores, POIs, buildings, gardens, waste bins, etc.) considered in the scenario and included in the area of interest, represented in JSON. Each entity is identified with a URI associated with an instance in the knowledge base;
A9. *roads* (geometry): a list of roads included in the area of interest, represented in GeoJSON, according to the formal model described in Section 3.2. Each road is identified with a URI associated with an instance in the knowledge base;
A10. *restrictions* (data structure): a list of traffic or access restrictions applied to entities and roads of the scenario, represented in JSON;
A11. *additionalData* (data structure): data required by specific analytics, represented in JSON;
A12. *processingStatus* (data structure): a list indicating the status of the scenario for each analytic used, represented in JSON. Each list entry can assume different values depending on the analytic to which it is referred;
A13. *operativeStatus* (string): a description indicating the status of the scenario; it can assume the following values: proposed, approved, and rejected;
A14. *version* (string): the version of the scenario used to implement a versioning system, with user-defined status labels. Please note that an automated versioning/evolution approach based on time was implemented using the dateObserved attribute;
A15. *dataObserved* (string): timestamps of the creation/modifications of the scenario, represented as string compliant with ISO 8601 [41].

Attributes A1–A5 describe the metadata of the scenario, responding to requirement R03. A6 is used to store the area of interest (R02), while in A7, the URI of the reference knowledge base is set (R04). To satisfy R05 and R06, attributes A8, A9, and A10 are used to store, respectively, the entities, the roads, and the restrictions included in the scenario, possibly modified. R07 and R11 are addressed with attribute A11, which is used to store possible additional data required by analytics or KPIs. A12 is used to track the processing steps reached with some analytics. Finally, A13, A14, and A15 are related to requirements R09 and R10 and consider different operative statuses and versions. 

To respond to requirement R08, in the next section, a formal method that assesses the validity of the (possibly modified) road graph included in a scenario is presented. 

### 3.2. Formal Road Graph Data Model

In this section, the formal representation of a road graph of a scenario model is presented to facilitate reasoning and formal verifications over the road graph, as well as to provide a formal framework to define KPIs involving the road graph and data connected through a knowledge base.

**Full road graph definition.** The full road graph (*FRG*) is defined as a tuple:

(1) FRG=<V, E, R, loc, dir, lanes, max_speed,restrictions, road>

where its elements are defined as follows:

- V is the set of nodes forming the road graph (i.e., the road junctions).
- E ⊂V×V is the set of edges of the road graph, where $\left(v,w\right)$ means that there is a physical link allowing one to go from node v to node w and vice versa.
- *R* is the set of roads.
- $loc:V\to R^{2}$ is a function associating a GPS position to each node.
- road:E→R is a function associating each edge to the road it belongs to.
- $dir:E\to \left\{any,+,-\right\}$ is a function stating for each edge $\left(v,w\right)\in E,$ the *direction* in which it can be traversed: any means it can be traversed both ways; +. Only from v to w; −. only from w to v.
- $lanes:E\to N^{+}$ is a function associating the number of lanes (>0) for each edge.
- $max\_speed:E\to R^{+}$ is a function that associates each edge with its max speed.
- $restrictions\subset E\times V\times E\times \left\{\begin{array}{c} no_{\leftarrow },\;no_{\to },no_{↑},\;no_{uturn},\;no_{exit}\\ only_{\leftarrow },only_{\to },\;only_{↑},\;only_{uturn} \end{array}\right\}$ models turn restrictions, where tuple (f, v,t, k) means that the restriction of type k applies to the edge f via node v to edge t; the v node has to be shared between edges f and t, for example, restriction $\left(\left(n_{1},v\right),v,\left(v,n_{2}\right),\;no_{\to }\right)$ means that from edge $\left(n_{1},v\right)$, it is not possible to turn to edge $\left(v,n_{2}\right)$.

The *FRG* is used to represent all the detailed road shapes, and from this, the Compact Road Graph (*CRG*) can be built, where edges connected in a sequence have the same associated data (*dir, road, lanes, max*_speed) and can be represented as a single edge. For this purpose, we introduced the following functions:

(2) $$prec_{E}\left(e\right)=\left\{e^{\prime }\in E\;\right|{\;\left(e^{\prime }\right)}_{2}={\left(e\right)}_{1}\}$$

(3) $$next_{E}\left(e\right)=\left\{e^{\prime }\in E\;\right|{\;\left(e\right)}_{2}={\left(e^{\prime }\right)}_{1}\}$$

where Equation (2) returns the set of edges that are insisting on edge e, while Equation (3) returns the set of edges that are next to e. Symbol ${\left(\cdot \right)}_{i}$ is a projection function used to obtain the *i*-th component of a tuple/sequence, i.e.,$\;{\left(\left(v,w\right)\right)}_{1}=v,\;{\left(\left(v,w\right)\right)}_{2}=w$, and ${\left(\cdot \right)}_{L}$ provides the last element of a sequence. Function ${\left(\cdot \right)}_{1}^{L-1}$ on a sequence provides the subsequence without the last element. 

**Compact road graph definition.** Given a *FRG* as in Equation (1), its *CRG* version is expressed as

(4) $$CRG=<V^{\prime },\;E^{\prime },\;R,\;loc,\;dir^{\prime },\;lanes^{\prime },\;max\_speed^{\prime },restrictions^{\prime },\;road^{\prime },M>$$

and it can be built by introducing an additional mapping function $M:E^{\prime }\to Seq(E)$ that maps each edge of the compact version to a sequence of edges of the full version, with the following constraints:

- $V^{\prime }\subset V$, the set of nodes of the compact version are a subset of the full version.
- $E^{\prime }\subset V^{\prime }\times V^{\prime }$ and $\forall e^{\prime }\in E^{\prime }.\;$
  ${{(e}^{\prime })}_{1}={{\left((M\left(e^{\prime }\right)\right)}_{1})}_{1}\wedge$ ${{(e}^{\prime })}_{2}={{\left((M\left(e^{\prime }\right)\right)}_{L})}_{2}\wedge .$
  $\forall e\in M\left(e^{\prime }\right).dir^{\prime }(e^{\prime })=dir\left(e\right)\wedge .$
  $lanes^{\prime }\left(e^{\prime }\right)=lanes\left(e\right)\wedge .$
  $\max\_speed^{\prime }\left(e^{\prime }\right)=\max\_speed\left(e\right)\wedge .$
  $road^{\prime }\left(e^{\prime }\right)=road\left(e\right)\wedge .$
  $\forall e\in {M\left(e^{\prime }\right)}_{1}^{L-1}.\left\|next_{E}\left(e\right)\right\|=1\wedge next_{E}\left(e\right)\subseteq M\left(e^{\prime }\right).$
- M *maps to the longest possible sequence of edges.*
- $restrictions^{\prime }=\left\{\left(f^{\prime },v,t^{\prime },k\right)\;\right|\;(f,v,t,k)\in restrictions\wedge f\in M\left(f^{\prime }\right)\wedge t\in M\left(t^{\prime }\right)\}.$

If in the *CRG*, there exist edges with only one next edge, i.e., $\left\{e^{\prime }\in E^{\prime }\right|$ $\left\|next_{E^{\prime }}\left(e^{\prime }\right)\right\|=1\}\neq \emptyset$, this means that there exists an edge on the road where the direction or the number of lanes or the max speed or the road name are changing.

Note that, with respect to a naïve graph modeling of the road network, the *FRG* or the *CRG* representations are required to take into account the possible restrictions. Then, to assess if a road graph (full or compact) is meaningful, some standard graph algorithms can be used to check, for example, the number of connected components or to find the shortest path between two nodes, since in a well-designed road network, all nodes should be reachable from all the other nodes.

## 4. Scenario Editor 

**Figure 2:**
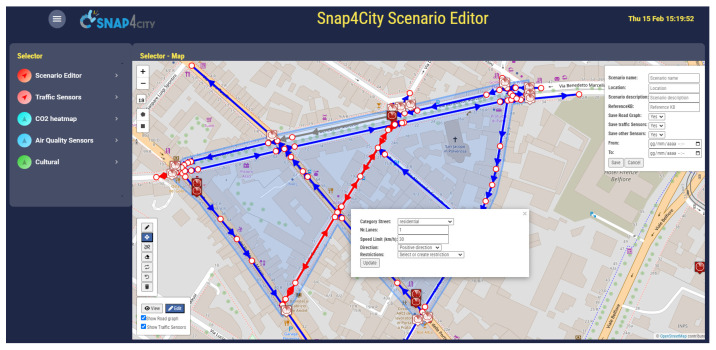
Snap4City scenario editor interface (in edit modality). Blue segments are singe direction roads; Red segments are roads open in both directions; Grey are closed roads.

The scenario editor was designed, developed, and enforced using Snap4City tools according to the requirements presented in Section 3 to provide a versatile and easy-to-use operator tool for defining, studying, and analyzing smart city scenarios [30]. In Figure 2, a screenshot of a Snap4City dashboard/tool including the proposed scenario editor is provided. As can be seen, a map is used as the background, while on the left and right sides of the map, panels and buttons are placed that can be used to create/edit the scenario. The save functionality is accessible to registered users, and the registration is free of charge.

The editor can work in editing and view modalities (see the button in the bottom left corner of the map): the former is the modality used to define a scenario and introduce changes, while the latter is used to display previously created scenarios, possibly delegated or made public. On the right side, a panel to specify scenario metadata is displayed, including fields for the scenario name, location, description, reference to a knowledge base, and start and end date-times of the scenario validity. In the top left corner, under the buttons to set the map zoom level, the button controls for drawing a squared or polygonal shape are provided. After having defined the shape of the area of interest, the road graph is retrieved from the knowledge base and shown over the map with interactive graphical elements. Roads are displayed with arrows specifying the direction and with different colors following the OSM classification: *Blue* is for roads with a single direction, *Red* for roads with bidirectional setting, *Yellow* for pedestrian pathways, *Cyan* for cycling paths, and *Grey* for closed roads.

Each road segment can be clicked on to access and modify specific information (category, number of lanes, max speed, direction, restrictions, etc.). In using the button in the bottom left corner, roads can be modified by creating, splitting, or deleting segments, dragging and joining nodes. Undo and redo actions were implemented to help the user in correcting possible errors. Entities are requested using the selector menu on the left side of the dashboard. Each selector entry specifies an entity kind (traffic, weather, air quality sensors, POIs, bus stops, etc.) that can be loaded independently into the scenario editor. Once the user completes the editing operations, the resulting scenario can be saved using the format described in Section 3.1.

Before saving the scenario, the system performs a consistency check on the defined road graph to highlight possible errors/inconsistencies using the formal model described in Section 3.2. In the cases in which the consistency check fails—e.g., when nodes with only entering/exiting roads are detected—an alert is sent to the user, and the different components are highlighted in the map. Additionally, a warning is presented to the user when the system detects roads having segments with different numbers of lanes or different max speeds along the same road. Even if this case is something that may happen in real cases, due to the relevance of such characteristics (e.g., a restriction), the system highlights suspicious cases to help the user avoid the introduction of errors in the scenario.

**Figure 3:**
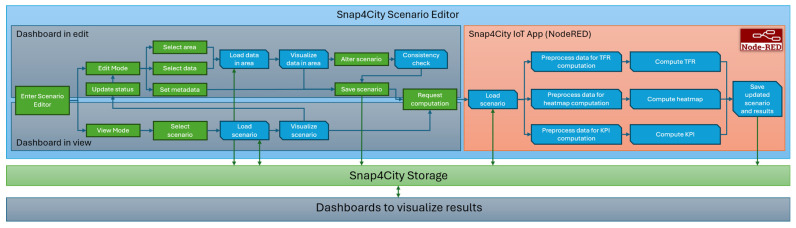
Scenario editing and usage workflow block diagram.

**Figure 4:**
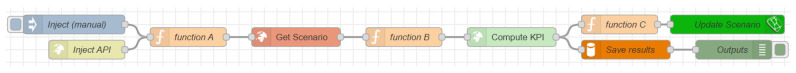
Example of Snap4City IoT App for KPI computation on based on a scenario.

In Figure 3, a block diagram of the workflow of the proposed scenario editor is shown. As can be seen, view and edit modalities are shown, and the sequence of steps that a user has to follow is visualized (the green block indicates user actions, and blue ones are automatic functions). Once a scenario is defined, IoT Apps (i.e., Node-RED flows) are available to execute analytic processes, such as the computation of the TFR, heatmaps, and KPIs. In Figure 4, an example of an IoT App used to compute KPIs in the context of a specific scenario is provided. The Node-RED flow can be manually activated by injecting a starting message in the flow, and/or by exploiting a HTTP endpoint (directly from a dashboard user interface), or through some events arriving on some broker. Then, in the Node-RED flow (IoT App), the recall of a specific scenario is prepared using function A, and the Get Scenario nodes retrieve the formal definition of the scenario via the Smart City API/MicroService. In the flow, the function B node is used to set up the input for the KPI computation carried out by the Compute KPI node. Such a node is a containerized R Studio or Python executor actionable through REST interfaces. R Studio and/or Python codes can be provided by the customers/developers. Finally, data analytic results can be saved in some storage, and the scenario can be updated after some data preparation is carried out in function C. The capability of saving the updated scenario enables the possibility to reload it to introduce further changes or set any additional input required by specific analytics. In Section 5, as a case study, the usage of the scenario editor for traffic flow reconstruction and a what-if analysis is presented.

### Scenario Editor Usability Test

**Figure 5:**
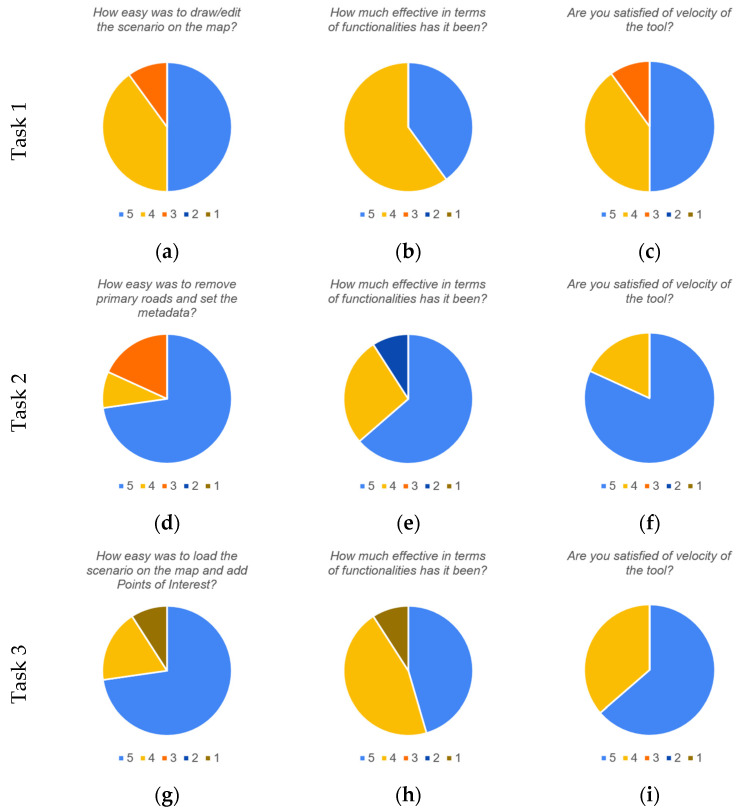
Pie charts reporting the usability test results. (**a**–**c**) show responses for the Task 1, for Question 1, 2, and 3 respectively. Similarly (**d**–**f**) are related to the three questions of Task 2. (**g**–**i**) show results for questions of Task 3. Questions are also reported on top of each chart.

To assess the usability and effectiveness of the proposed scenario editor, a usability test was carried out. We built a dashboard including the scenario editor with a Google form embedded as external content [41]. Upon accessing the dashboard, a video tutorial is available that illustrates the editor functionalities, and the instructions followed to perform the test are reported. Several users with different backgrounds (from data analysts to urban and mobility planning experts) were asked to complete three tasks and respond to a series of questions expressing their votes on a Likert scale from 1 (not satisfied) to 5 (very satisfied) and optionally provide some comments. In the first task, the users were asked to draw an area of interest describing the scenario and make some changes. Then, three questions were asked: *How easy was to draw/edit the scenario on the map?*; *How much effective in terms of functionalities has it been?*; *Are you satisfied with the velocity of the tool?* Regarding the first question, 50% of the users expressed maximum satisfaction with a vote of 5, 40% voted 4, and 10% voted 3. In the second question, 40% of users gave a vote of 5, and 60% voted 4. In the final question, 50% of the users opted for a 5, 40% gave a 4, and 10% voted 3. After the first task, some users expressed some difficulties at first sight, and after watching the 10 min video tutorial, they were able to carry out the tasks. In the second task, the users were asked to continue to work on the previously defined scenario, hide the primary roads, and set the scenario metadata. The following questions were asked: *How easy was to remove primary roads and set the metadata?*; *How much effective in terms of functionalities has it been?*; *Are you satisfied with the velocity of the tool?* For the first question, 73% of users gave a 5, 9% voted 4, and 18% voted 3. In the second question, 64% voted 5, 27% gave a 4, and 9% voted 2. For the third question, 82% voted 5, and 18% opted for a 4. Comments on the second task suggested to alphabetically order the road filters and provide some support in filling in the metadata. Finally, in the third task, users were asked to load a scenario and add POIs on the map using the functionalities of the tools. The questions posed were as follows: *How easy was to load the scenario on the map and add Points of Interest?*; *How much effective in terms of functionalities has it been?*; *Are you satisfied with the velocity of the tool?* In the first question, 73% of users opted for a 5, 18% voted 4, and 9% gave a 1. In the second question, a 5 was given by 46% of users, 45% voted 4, and 9% gave a 1. For the third question, 64% of votes were a 5, and 36% of users voted 4. In Figure 5, pie charts of the usability test results are shown. Even though some improvements were requested, such as the introduction of additional functionalities and minor corrections to the interface to augment its accessibility, overall, the usability test obtained very positive feedback from the users with 93% of votes expressed as 5s and 4s. For completeness, in Table 3, the averages of the received votes (with respective standard deviations) are reported for each question. As can be seen, all the questions obtained average votes higher than 4.

## 5. Case Study: Traffic Flow Reconstruction

Understanding the evolution of traffic within urban environments is important for effective city planning and management to, for example, face the challenges posed by growing populations and increasing vehicular density, or to address events and planned activities on the urban infrastructure. Accurate insights into how traffic patterns evolve over time enable decision makers to implement strategic measures that enhance mobility, reduce congestion, and overall improve the urban resilience. In this context, the TFR case study presents a comprehensive approach contributing to informed decision making. Using the scenario editor and the data-driven tools, a user can analyze and reconstruct traffic flows in the area of interest and perform what-if analyses.

The proposed scenario editor can be used to select a specific area of the map, retrieve information about the road network and traffic sensors, and possibly apply changes, defining a new scenario for traffic analysis. The scenario editor allows the users to choose which sensors to consider for the traffic reconstruction procedure, or to assign some reference time trends. The computation of the TFR is carried out by exploiting the algorithm presented in [33] that is based on the solution of a fluid dynamic problem based on partial differential equations (PDEs). As usual, for solving PDE problems, boundary conditions must be specified. In this case, the reconstruction method requires knowing the traffic flow entering/exiting the area of interest. For this reason, the scenario editor automatically detects road sections that intersect the borders of the area of interest. On these points, virtual traffic sensors are placed. In using such virtual sensors, the user can specify 24 h trends to be used as constraints to be satisfied (to maintain the origin destination needs of the area at the borders). Different kinds of traffic time trends can also be exploited as typical time trends generated from historical traffic data, predicted typical time trend data, or arbitrary time trends defined by the user. 

Once the editing process is completed, the scenario is saved according to the defined data model (see Section 3.1), with the scenario *processingStatus* attribute set to *init*. Then, the user can load the scenario in the TFR analytic tool, and a first pre-processing phase can be carried out. Road intersections connected to only two road segments with the same number of lanes and max speed are removed, and related road segments are merged, passing from a *FRG* to a *CRG* (see Section 3.2), which are two different graph representation modalities of the same scenario. This reduces the fragmentation of the road network, helping in the required discretization of the numerical solution (see [33] for further details). After this phase, the scenario is updated: attribute A9 now describes the *CRG*, and the *processingStatus* moves to *merged*. 

Then, additional inputs are requested from the user. The scenario is loaded into the editor, and the user is asked to set the road segment capabilities (i.e., an estimate of the number of vehicles that a road can accommodate). Such information is exploited to compute so-called Traffic Distribution Matrices (TDMs) that represent how vehicles are distributed at road junctions. More precisely, ${TDM=\{w_{ji}\}}_{j=n+1,\ldots ,n+m,i=1,\ldots ,n}$ such that $0<w_{ji}<1$ and $\sum_{j=n+1}^{n+m}w_{ji}=1$, for i=1,…,n and j=n+1,…,n+m, where $w_{ji}$ coefficients (called weights) are the percentages of vehicles arriving from the i-th incoming road and taking the j-th outcoming road (assuming that, on each junction, the incoming flux coincides with the outcoming flux). The scenario is saved again, with road capabilities and the TDM saved in the *additionalData* attribute (A11), and the *processingStatus* set to *ready*. Now the TFR can run on the updated scenario to compute the traffic reconstruction. 

To summarize, firstly, the user defines the scenario in the *init* version. Then, a merging process is carried out to pass from microsegments to merged roads, moving the scenario state to *merged*. The scenario is loaded in the editor and the user specifies the segment capabilities (and indirectly, the weights of the TDM), moving the scenario state to *ready*. Finally, the algorithm for computing the TFR is executed, producing for each road, a traffic density.

To assess the correctness and the validity of the proposed approach, two tests were carried out. In the first case, we studied the correspondence between a TFR computed on a wide road network, i.e., the whole city of Florence in Italy (macro scale), and the reconstruction obtained on a small sub-graph (micro/meso scale) according to the area of interest delimited by a scenario. The assessment consisted in evaluating differences between the two cases of the estimated traffic flow (from macro and micro scale) in each road segment of the graph (micro scale) and at the borders of the scenario (see Section 5.1). 

Then, in a second validation case, we assessed the impact of alterations in the road graph by changing road travel directions to create additional paths to reduce congestion on the principal roads (see Section 5.2), keeping the constraints at the border fixed, and maybe as well in some control points if requested.

### 5.1. Consistency and Correctness of TFR

**Figure 6:**
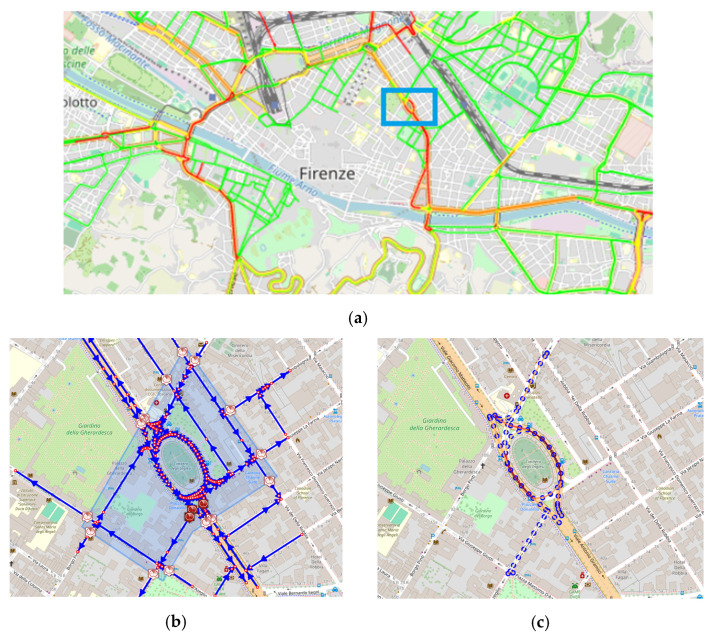
Consistency test. In (**a**), the TFR computed on the entire city of Florence at the macro scale, $R_{H}$. Road colors indicate the level of congestion: green for FreeFlow, yellow for FluidFlow, orange for HeavyFlow, and red for VeryHeavyFlow. The blue rectangle represents the selected area for the comparison. In (**b**), the specific area delineated using the scenario editor, corresponding to the blue rectangle in (**a**), with in blue the roads, in red the road junctions. In (**c**), the TFR obtained in the micro-scale sub-graph, $R_{S}$, for the matching segments with $R_{H}$ in the delineated area. Reconstruction corresponds to 9:00 a.m. on 28 September 2023. As in (**a**) colors correspond to congestion levels.

A first analysis was performed to assess the correctness of the local traffic flow reconstructions. A small area of the city of Florence (see Figure 6a) was selected as a scenario to test if the TFR results on the micro scale are consistent with those extrapolated from the reconstruction computed in [33] on the macro scale. The TFR computed on the 28 September 2023, referred as $R_{H}$, was chosen for the analysis. In using the scenario editor, the area of interest was selected (see Figure 6b). For the area of interest, the scenario fetched the road elements on which the reconstruction $R_{H}$ was performed. The virtual sensors were considered to reproduce the same boundary conditions; therefore, they were initialized with the flow values of $R_{H}$. The traffic sensors inside the scenario were also considered, and the same TDMs were imposed to obtain the small-scale reconstruction for the defined scenario, referred to as $R_{S}$. The reconstruction at 9:00 a.m. on 28 September 2023 is shown in Figure 6c.

**Figure 7:**
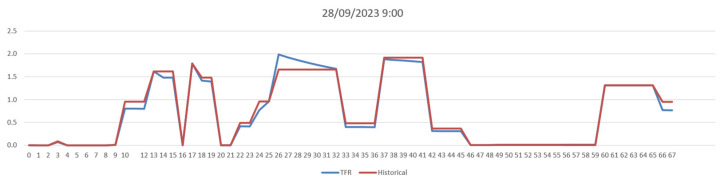
Consistency test. Comparison of the traffic flow reconstruction (red line) from macro scale and the TFR (blue line) only on the scenario area of the 68 segments at 9:00 on 28 September 2023.

The TFR algorithm produced almost identical reconstruction results on the defined micro-scale scenario with respect to the macro scale, showing an equal level of congestion on the corresponding road elements. Figure 7 shows a comparison between the historical traffic flow values computed on the macro scale and those obtained in the scenario micro scale, at 9:00 a.m. on 28 September 2023, for 68 road segments of 20 m. The reconstructions show a high degree of correspondence. On average, a mean absolute error of 0.05648 [cars/20 m] was achieved, demonstrating the robustness of the TFR method and the validity of the proposed approach passing from macro- to micro-scale computation. For reference, the mean flow value of $R_{H}$ was 0.734774 (cars/20 m), and the error achieved was about 6.81%. Note that we could not achieve perfectly identical results since in $R_{S}$, the selected sub-graph generated a different merged graph with respect to $R_{H}$. As a consequence, the reconstructions worked on slightly different road segments, preventing a perfect match between the $R_{H}$ and $R_{V}$ road graphs.

### 5.2. What-If Analysis for Traffic Congestion Reduction

**Figure 8:**
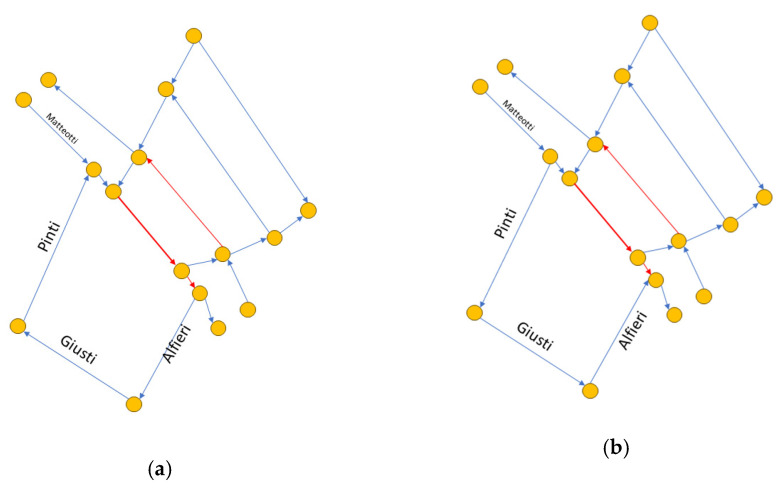
Graph structures of the road network in the selected area: (**a**) graph of $v_{1}$; (**b**) graph of $v_{2}$.

In this second study, we exploited the scenario editor to perform a what-if analysis on traffic flows. In a first test, the chosen area was the same on which the consistency and correctness analyses were performed (reported in Figure 6b). This version is referred to as version $v_{1}$, and its graph structure is shown in Figure 8a. This area is often congested in the main roads, and in Figure 8, these road sections are highlighted in red. Measurements for real traffic sensors encompassed in the area, and for the virtual sensors on the border, were sampled from historical data. No changes were introduced to the road network. With the aim of improving the traffic flow in the selected area using the editor, it was successfully possible to verify the effects of an alternative road network configuration. Version $v_{2}$ was created by inverting the travel directions for streets Borgo Pinti, via Giuseppe Giusti, and via Vittorio Alfieri. The new path should alleviate the traffic flow moving from north to south, redirecting part of the traffic onto the alternative path. The graph structure of $v_{2}$ is shown in Figure 8b. The TDM value related to Matteotti was set to 70, and that of Pinti, to 12. This means that, in percentage, 14.63% of cars should take the new Pinti route, while the remaining 85.36% should continue the roundabout, according to the weight coefficients in the TDM. To complete the analysis, a third version ${(v}_{3})$ was considered by taking $v_{2}$ and changing the TDM value of Pinti from 12 to 25. Such a change was made to answer t the question: what happens if more cars choose the novel route? In detail, we wanted to observe what would happen if, instead of around 15%, the percentage of vehicles choosing Pinti was 26.32%, with an increase of about 10%.

**Figure 9:**
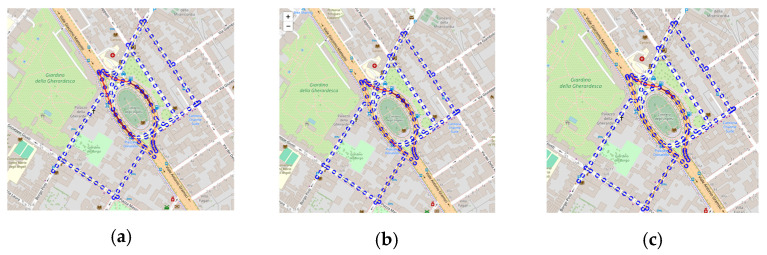
What-if analysis for TFR. In (**a**), the current scenario ($v_{1}$). In (**b**), a new scenario with a novel traffic route obtained by inverting the travel directions of three roads ($v_{2}$). In (**c**), the same road network used in $v_{2}$ but with different TDMs ($v_{3}$).

According to the what-if analysis, we tested if the new configuration could improve the traffic congestion in the selected area. The TFRs for $v_{1}$, $v_{2}$, and $v_{3}$ were computed, and the results are reported in Figure 9. As can be seen, by having created a new route, the congestion on the main roads decreased with respect to $v_{1}$. However, from the graphs in Figure 9, it is possible to note that in $v_{2}$, there was still a 20 m segment of a heavy traffic flow state (represented in red); meanwhile, in $v_{3}$, no congestion was visible on the main road.

To quantify the improvements, we divided the road segments into four groups, depending on the measured traffic density ρ in the initial scenario with respect to the critical density $\rho_{C}$ (equal to half the maximum density, i.e., $\rho_{MAX}=2\rho_{c}$): *FreeFlow*, if $0\leq \rho <\frac{1}{2}\rho_{C}$; *FluidFlow*, if $\frac{1}{2}\rho_{C}\leq \rho <\rho_{C}$; *HeavyFlow*, if $\frac{1}{2}\rho_{C}\leq \rho <\frac{3}{4}\rho_{C}$; *VeryHeavyFlow*, if $\frac{3}{4}\rho_{C}\leq \rho <\rho_{MAX}$. Note that traffic flow f and density ρ are related according to the following equation:

(5) $$f=v_{MAX}\left(1-\frac{\rho }{\rho_{MAX}}\right)\rho$$

where $v_{MAX}$ is the maximum velocity. For each class, the percentage of segments belonging to that class is considered and represented with *FRrs*, *FLrs*, *HErs*, and *VHrs*, respectively, for the *FreeFlow*, *FluidFlow*, *HeavyFlow*, and *VeryHeavyFlow* classes. 

**Figure 10:**
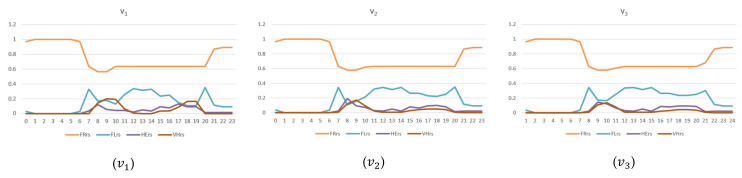
A 24 h TFR comparison of the different segment states: *FreeFlow (FRrs)*, *FluidFlow (FLrs)*, *HeavyFlow (HErs)*, and *VeryHeavyFlow (VHrs)*.

In Figure 10, the traffic densities for the 24 h in the four groups for $v_{1}$, $v_{2}$*,* and $v_{3}$ are reported. The trends reveal the decrease in the number of road segments in the *VeryHeavyFlow* status (*VHrs*) from $v_{1}$ to $v_{2}$ and $v_{3}$. However, the differences between $v_{2}$ and $v_{3}$ are less evident. The results are also reported numerically in Table 4, showing the average values (h24) for the different states in the different versions of the scenario considered. A major improvement can be seen in the percentage of segments in the *FluidFlow* state (*FLrs*) with respect to $v_{1}$, $v_{2},\;and\;v_{3}$ rising from to 0.1501 to 0.1705 and 0.1744, respectively. Even if the percentage of segments in the *HeavyFlow* state (*HErs*) slightly increases in the modified versions, this is acceptable since it determines an important decrease in the segments in the *VeryHeavyFlow* state (*VHrs*) from 0.0455 in $v_{1}$ to 0.0274 in $v_{2}$ and 0.0223 in $v_{3}$. 

To evaluate the overall improvement in the traffic in the defined scenarios, specific delta metrics for the different traffic density states were defined as follows:

- $\delta_{FRrs_{1,2}}=FRrs_{v1}-FRrs_{v2},$ $\delta_{FRrs_{1,3}}=FRrs_{v1}-FRrs_{v3};$
- $\delta_{FLrs_{1,2}}=FLrs_{v1}-FLrs_{v2}$,${\;\delta }_{FLrs_{1,3}}=FLrs_{v1}-FLrs_{v3};$
- $\delta_{HErs_{1,2}}=HErs_{v1}-HErs_{v2}$, $\delta_{HErs_{1,3}}=HErs_{v1}-HErs_{v3};$
- $\delta_{VHrs_{1,2}}=VHrs_{v1}-VHrs_{v2}$,$\;\delta_{VHrs_{1,3}}=VHrs_{v1}-VHrs_{v3}.$

Please note that if $\delta_{FRrs}$ and $\delta_{FLrs}$ are positive, then the number of segments (in percentage) with uncongested traffic states decrease upon passing from the original scenario to the modified one. Generally, the occurrence of a positive value in $\delta_{FRrs}\;$(and $\delta_{FLrs})$ indicates a negative effect. Thus, when $\delta_{FRrs}$ and $\delta_{FLrs}$ are negative values, the traffic state is improved between the compared scenarios. This means that the segments in the uncongested traffic state increase upon passing from the original scenario to the modified one. Vice versa, if $\delta_{HErs}$ and $\delta_{VHrs}$ are positive, then the congested traffic state decreases in the compared scenarios. To evaluate the percentage of improvement according to the *FreeFlow*, *FluidFlow*, *HeavyFlow*, and *VeryHeavyFlow* traffic states, we have to take into account a multiplicative factor $m_{c}$ according to the above dissertation. Then, $m_{c}=-1$ for the cases of the *FRrs* and *FLrs* estimations, and $m_{c}=1$ when *HErs* and *VHer* are considered. For example, in order to estimate the percentage of improvement of scenario $v_{2}$ with respect to the original one, we consider the following formula for *FRrs*, where $m_{c}=$ −1:

(6) $$PercentageOfImprovement_{FRrs_{1,2}}=\frac{m_{c}*\delta_{FRrs_{1,2}}}{FRrs_{v1}}*100$$

Similarly, different states were evaluated, and the related estimations are reported in Table 5. The results demonstrate significant reductions in the number segments in the *VeriHeavyFlow* state (*VHrs*) of 39.86% and 50.98% upon passing from scenario $v_{1}$ to $v_{2}$ and $v_{3},$ respectively, at the expense of a marginal increment in segments in the *FreeFlow* and *HeavyFlow* states. However, such negative impacts are negligible compared to the benefits obtained that significantly reduce the overall congestion, particularly on the main roads.

**Figure 11:**
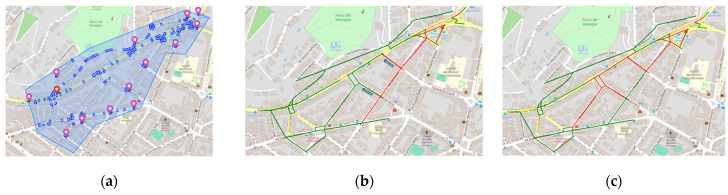
Scenarios and TFRs of the second experiment. TFRs referred to 9.00 a.m. (**a**) Defined scenario $v_{4}$. (**b**) TFR of $v_{4}$. The modified scenario $v_{5}$ was defined by inverting the travel directions of two roads according to the cyan arrows in (**b**). (**c**) Updated TFR computed on $v_{5}$.

To better illustrate the potentiality of the proposed scenario editor, the results on a different area are reported in the following. Figure 11a shows an area where the new scenario ($v_{4}$) was defined. Boundary conditions for the incoming roads were set using a typical time trend on the basis of historical flow data, and TDMs were set according to the road type, where higher turn probabilities were assigned to main roads. In Figure 11b, the original TFR is shown, with roads colored according to the respective flows (green, yellow, orange, and red, respectively, for the *FreeFlow*, *FluidFlow*, *HeavyFlow*, and *VeryHeavyFlow* traffic states). Suppose an operator wants to reduce the congestion on the main road, which is presently in the *VeryHeavyFlow* state (i.e., Via dei Carioli). To achieve such a result, the directions of two auxiliary roads are inverted (indicated with cyan arrows in Figure 11b) to offer alternative routes to the vehicles, defining a novel scenario version, $v_{5}$. The system computed the TFRs for $v_{5}$, and the results are presented in Figure 11c and Table 6. As it can be observed, the introduced changes provoked a negative impact: the percentage of segments in the *FreeFlow* state (*FRrs*) decreased from 0.8071 to 0.7293, while the percentage of segments in the *VeryHeavyFlow* state (*VHrs*) increased from 0.1264 to 0.1961. Even if the congestion was successfully reduced on most of the main road, the changes introduced lead to a series of cascading effects that worsen the general traffic conditions on several neighbor streets. 

Our scenario editor resulted to be a valid solution for assessing the impact of changes in the urban environment, providing clear insights for assessing their validity and usefulness and highlighting effects that could not be easily foreseen.

Finally, in Table 7, the execution times for the three scenarios are reported. Computations were carried out on a virtual machine on the cloud, with 16 GB of RAM on a CPU only. As can be seen, the TFR computation for 24 h took an average of about 146 s, with slight differences among the two experiments due to different road network dimensions and complexities.

## 6. Conclusions

In this paper, a novel scenario model and editor have been presented. The solution was designed according to an analysis of a large range of smart city requirements needed to cover relevant cases in terms of computing scenario-based metrics, KPIs, heatmaps, TFRs, etc. This is a fundamental feature since city officers and decision makers must face challenges spanning multiple domains, not limited to traffic. Unlike other state-of-the-art solutions that lack data integration and are focused on traffic analysis and provide limited versioning capabilities, the proposed solution responds to all the requirements identified, reported, and discussed in this paper. Our solution implements standard data models and a formal method analysis that can be integrated with multiple analytics spanning different domains and allows the users to load and visualize the road network and other entities in a specific area and then to alter the current status to define multiple what-if scenarios. Moreover, our scenario editor is a web-based application released as open-source, does not require any installation, and offers high levels of reliability, accessibility, security, and privacy thanks to its integration into the Snap4City platform. Such a solution can undoubtfully help city councils and decision makers in planning and development activities by assessing the current urban status and performing simulations on multiple scenarios after changing specific elements of the urban context. A usability test was carried out, collecting positive feedback from several users. Case studies were presented, involving the production of scenarios for traffic congestion analyses and KPI assessments, showing how the proposed solution could be effectively used. Thanks to the scenario formalization, which can represent any scenario dimension with the defined NGSIv2 model, the system is able to pass from macro- to micro-scale computations without loss of generality. This was demonstrated in the case study where the reconstruction analytics, originally used for a macro-scale analysis, produced consistent results on the micro scale. This also enables the use of other analytic software, already implemented and accessible in the Snap4City open-source platform, to realize predictions, simulations, heatmaps, OD matrices, and reconstructions (according to the selected input data) to assess the impact of changes in different domains (e.g., mobility, energy, environment) in terms of the different KPIs and metrics possible. Therefore, the proposed solution can support city operators and researchers to address different challenges by performing analyses on the current state to find critical conditions, and it can be used to carry out what-if analyses to assess the impact of possible changes and make informed decisions for urban planning strategies. The scenario editor is accessible and can be tested on Snap4City.org. It is presently in use for research and development activities in the context of the CN MOST, the National Center on Sustainable Mobility in Italy. 

## Figures and Tables

**Table 1:** Scenario editor requirements.

ID	Name	Description of the Functional Requirements of a Scenario Editor
R01	Map visualization and controls	Show/select ground map to be used as the main canvas over which the user can define and study the scenario. Controls to move and zoom in on the map must be provided, with the possibility of changing the ground map when needed. The map is a visual representation of the geo information, for the minimal case, the graphs of roads, their relationships, and details.
R02	Area of interest definition	Draw/change a polygonal shape of arbitrary size to define the area of interest of the scenario, selecting a portion of the map and of the corresponding geo information. The scenario could be composed of multiple disjoint areas.
R03	Metadata setting	Set some metadata describing the scenario, such as its name, a description, the temporal validity (from date time to date time), the author, a purpose, etc.
R04	Knowledge base management	Work on different maps and geo information, which can be coded into different knowledge bases or other storages, to fetch the geolocated information (i.e., entities and roads) to be taken into account in the scenario.
R05	Road graph selection and management	Manage the road graph; each road segment must be visualized and managed in the scenarios. The road segments may present a number of descriptive characteristics, such as, type, travel direction, presence of restrictions, lanes, sidewalks, parking lots, etc. Each road must be selectable by the user to access additional information, such as name, type, length, number of lanes, maximum speed, etc. The manipulation of the road graph must be possible, for example, add, remove, or alter a road, invert the travel direction, increase or reduce the number of lanes, etc. In the representations of road segments, visual coding should be used to provide information at a glance.
R06	Entity selection and management	Manage geolocated entities such as IoT devices with time series data (such as semaphores, sensors/actuators, waste bins, parking sensors, luminaries, Wi-Fi access points, tv cameras, parking in structures); urban furniture (such as pedestrian crossing, benches, flowerbeds, fountains for drinkable water, toilets); and POIs (such as banks, cultural services, schools, commercial areas, restaurants, hotels). They must be visualized over the map upon user request. Each entity must be selectable by the user to inspect additional information (i.e., metadata, position, real-time and/or historic data, etc.). The manipulation of entities must be permitted, for example, to disable/enable an IoT device, select the measurements of interest, choose between real-time, historic, predicted, typical time trend data, change the semaphore timings, move a pedestrian crossing, etc.
R07	Enabling analytic computation	Define the context on which one could apply a large number of analytical processes including for example, the computation of traffic flow reconstructions, environmental analysis, environmental heatmaps, 15 min city index, KPIs to quantify some analysis, semaphore analysis, etc. For each analytic, the user has to be capable of composing the scenario and composing different inputs. This is the basis on which to enable the usage of the scenario for what-if analysis, exploiting several scenarios that must be inspected to verify their validity for solving a specific case.
R08	Validation through the activation of consistency analysis	Validate the scenario by means of one or a set of methods to assess its consistency and completeness in terms of road graph, entities, metadata, etc. The validation process has to involve an in-depth spatial analysis of the road graph as well as the compatibility check among the selected inputs.
R09	Scenario evolution over time	A scenario can evolve over time in terms of operative status (e.g., proposed, accepted, rejected), processing status (e.g., init, runnable, completed), and version. Each step must be related to a specific timestamp.
R10	Scenario management	To create a new scenario, save the defined scenario, load a previously created scenario, and save it again, possibly with a different name, etc.
R11	Models and custom	Scenario should be conformant to a model, on which additional variables can be added.

**Table 2:** Comparison of compliance of scenario editors to defined requirements.

Req.	GIS [19,20]	OSM iD Editor [24]	SUMO Netedit [28]	PTV [26,27]	Snap4City
R01	Yes	Yes	Yes (limited)	Yes (limited)	Yes
R02	No	No	No	No	Yes
R03	No	No	Yes	Yes	Yes
R04	Yes	No	Yes	Yes	Yes
R05	Yes	Yes	Yes	Yes	Yes
R06	Yes (no real-time)	Yes (no real-time)	Yes (no real-time)	Yes (no real-time)	Yes
R07	No	No	Yes (traffic only)	Yes (traffic only)	Yes
R08	No	Yes	Yes (partial)	Yes	Yes
R09	Yes (manual)	Yes (changelog)	Yes (manual)	Yes	Yes
R10	Yes	Yes	Yes	Yes	Yes
R11	No	Yes	Yes	Yes	Yes

**Table 3:** Averages (AVG) and standard deviations (STD) of the votes obtained for each question of the usability test of the scenario editor.

Task	Question	AVG	STD
1	*(a) How easy was to draw/edit the scenario on the map?*	4.4	0.7
	*(b) How much effective in terms of functionalities has it been?*	4.4	0.5
	*(c) Are you satisfied with the velocity of the tool?*	4.4	0.7
2	*(d) How easy was to remove primary roads and set the metadata?*	4.5	0.8
	*(e) How much effective in terms of functionalities has it been?*	4.5	0.9
	*(f) Are you satisfied with the velocity of the tool?*	4.8	0.4
3	*(g) How easy was to load the scenario on the map and add Points of Interest?*	4.5	1.2
	*(h) How much effective in terms of functionalities has it been?*	4.2	1.1
	*(i) Are you satisfied with the velocity of the tool?*	4.6	0.5

**Table 4:** Average percentages of road segments in the four groups, *FreeFlow*, *FluidFlow*, *HeavyFlow*, and *VeryHeavyFlow*, in 24 h for the three scenario versions.

Scenario Version	*FRrs* (FreeFlow)	*FLrs* (FluidFlow)	*HErs* (HeavyFlow)	*VHrs* (VeryHeavyFlow)
$v_{1}$	0.7649	0.1501	0.0396	0.0455
$v_{2}$	0.7607	0.1705	0.0414	0.0274
$v_{3}$	0.7622	0.1744	0.0411	0.0223

**Table 5:** Reduction values regarding the amount of road segments in the different traffic states used to evaluate the percentage of reduction between the different scenarios.

Delta	Value	Percentage of Reduction
$\delta_{FRrs_{1,2}}$	0.00416	−0.54%
$\delta_{FRrs_{1,3}}$	0.00267	**−0.35%**
$\delta_{FLrs_{1,2}}$	−0.0205	13.69%
${\;\delta }_{FLrs_{1,3}}$	−0.0244	**16.27%**
$\delta_{HErs_{1,2}}$	−0.0017	−4.51%
$\delta_{HErs_{1,3}}$	−0.0014	**−3.76%**
$\delta_{VHrs_{1,2}}$	0.0181	39.86%
$\delta_{VHrs_{1,3}}$	0.0232	**50.98%**

**Table 6:** Average percentages of road segments in the four groups, *FreeFlow*, *FluidFlow*, *HeavyFlow*, and *VeryHeavyFlow*, in 24 hours for the second experiment on scenarios $v_{4}$ and $v_{5}$.

Scenario Version	*FRrs* (FreeFlow)	*FLrs* (FluidFlow)	*HErs* (HeavyFlow)	*VHrs* (VeryHeavyFlow)
$v_{4}$	0.8071	0.0414	0.0251	0.1264
$v_{5}$	0.7293	0.0569	0.0174	0.1961

**Table 7:** Computational times (in seconds).

Scenario Version	Computational Times (s)
$v_{1}$	161.259
$v_{2}$	162.330
$v_{3}$	159.402
$v_{4}$	120.368
$v_{5}$	126.924

## Data Availability

Data are contained within the article.
